# Nrf2 attenuates inflammatory response in COPD/emphysema: Crosstalk with Wnt3a/β‐catenin and AMPK pathways

**DOI:** 10.1111/jcmm.13628

**Published:** 2018-04-16

**Authors:** Wenhui Cui, Zhihui Zhang, Panpan Zhang, Jiao Qu, Cheng Zheng, Xiaoting Mo, Wencheng Zhou, Liang Xu, Hongwei Yao, Jian Gao

**Affiliations:** ^1^ First Affiliated Hospital of Anhui Medical University Hefei Anhui China; ^2^ Second Affiliated Hospital of Dalian Medical University Dalian Liaoning China; ^3^ General Hospital of Datong Coal Mining Group Datong Shanxi China; ^4^ School of Pharmacy Dalian Medical University Dalian Liaoning China; ^5^ Department of Critical Care and Pulmonary Medicine Shanxi Medical University Second Hospital Taiyuan Shanxi China

**Keywords:** AMP‐activated protein kinase, chronic obstructive pulmonary disease, cigarette smoke, inflammation, nuclear factor erythroid‐2 related factor‐2, Wnt/β‐catenin

## Abstract

Chronic obstructive pulmonary disease (COPD) is characterized by persistent airflow limitation and abnormal inflammatory response. Wnt/β‐catenin and AMP‐activated protein kinase (AMPK) have been shown to modulate lung inflammatory responses and injury. However, it remains elusive whether Wnt/β‐catenin and AMPK modulate nuclear factor erythroid‐2 related factor‐2 (Nrf2)‐mediated protective responses during the development of emphysema. Here we showed that treatment with a Wnt pathway activator (LiCl) reduced elastase‐induced airspace enlargement and cigarette smoke extract (CSE)‐induced lung inflammatory responses in WT mice, which was associated with increased activation of Nrf2 pathway. Interestingly, these effects of LiCl were not observed in Nrf2^−/−^ mice exposed to elastase. In normal human bronchial epithelial (NHBE) cells, Wnt3a overexpression up‐regulated, whereas Wnt3a knockdown further down‐regulated the levels of Nrf2 and its target proteins heme oxygenase‐1 (HO‐1) and NAD(P)H: quinone oxidoreductase 1 (NQO1) by CSE treatment. In contrast, Nrf2 deficiency did not have any effects on Wnt/β‐catenin pathway in mouse lungs and NHBE cells. Both elastase and CSE exposures reduced AMPK phosphorylation. A specific AMPK activator metformin increased Wnt3a, β‐catenin, Nrf2 phosphorylation and activation but reduced the levels of IL‐6 and IL‐8 in NHBE cells and mouse lungs exposed to CSE. Furthermore, Nrf2 deficiency abolished the protection of metformin against CSE‐induced increase in IL‐6 and IL‐8 in NHBE cells. In conclusion, Nrf2 mediates the protective effects of both Wnt3a/β‐catenin and AMPK on lung inflammatory responses during the development of COPD/emphysema. These findings provide potential therapeutic targets for the intervention of COPD/emphysema.

## 1 INTRODUCTION

Chronic obstructive pulmonary disease (COPD) is a chronic lung disease, which is characterized by a progressive airflow obstruction along with abnormal inflammatory responses (bronchiolitis) and alveolar destruction (emphysema).^1^ Cigarette smoking (CS) is the main aetiological factor for the development of COPD. CS contains various toxic chemicals including reactive aldehydes, quinones and semiquinones in puff.^2^ CS also causes endogenous reactive oxygen species release from recruited macrophages and neutrophils, which further induces inflammatory responses and subsequent lung injury. Therefore, the modulation for redox balance plays pivot roles in regulating inflammatory response, which could be therapeutic targets for COPD/emphysema treatment. However, there is still lack of full understanding of molecular mechanisms underlying abnormal inflammatory responses in COPD.

Wnt/β‐catenin is an evolutionarily conserved cellular signalling system that involves in embryonic development, homoeostatic self‐renewal and pathogenesis of human diseases.^3^ In the absence of Wnt signals, β‐catenin is phosphorylated and degraded by destruction complex including Axin, adenomatous polyposis coli (APC) and glycogen synthase kinase (GSK)‐3β. Upon Wnt activation, β‐catenin translocates to the nucleus and then associates with TCF/LEF family transcription factors to activate target gene expression. Wnt/β‐catenin has been shown to increase proliferation of lung epithelial cells during lung epithelial injury and repair.^4^ Furthermore, Wnt3a activation can inhibit neutrophils influx into the alveolar airspace of injured lungs as well as experimental emphysema.^5^ However, the molecular mechanisms underlying the protection of Wnt signal against COPD are unknown.

Nuclear factor erythroid‐2‐related factor‐2 (Nrf2) is a master transcription factor, which protects against oxidative stress by inducing the expression of anti‐oxidative genes including NQO1 and HO‐1. It is well‐known that Nrf2 protects against the development of emphysema in rodents.^6^, ^7^, ^8^, ^9^ A recent study demonstrates that Wnt3a stabilizes Nrf2 by preventing its GSK‐3‐dependent phosphorylation and degradation in hepatocytes.^10^ Furthermore, AMP‐activated protein kinase (AMPK) has been shown to activate Nrf2, which links to GSK‐3β inhibition.^11^, ^12^ However, it is unknown whether Nrf2 mediates the protection of Wnt3a and AMPK against pulmonary emphysema. We hypothesize that Wnt3a/β‐catenin and AMPK co‐regulate Nrf2, thereby modulating lung inflammatory and injurious responses during the development of COPD/emphysema.

## 2 MATERIALS AND METHODS

### 2.1 Mice and drug administration

Nrf2^−/−^ mice with C57BL/6J background were kindly provided by the Jiangsu Province Institute of Traditional Chinese Medicine, Nanjing, China, which were originally purchased from the Jackson Laboratory, USA (Order number:3363093). These mice were bred by crossing Nrf2^−/−^ and C57BL/6J wild‐type (WT) mice, and maintained with free access to water and food in the laboratory of the Animal Center of Anhui Medical University, Hefei, China with a 12:12 light–dark cycle, 35% humidity. Age‐matched Nrf2^−/−^ mice and WT littermates were intratracheally injected with porcine pancreatic elastase (PPE, 80 U/kg body weight, SLBH3907V; Sigma, Munich, Germany) once to induce pulmonary emphysema.^13^ Same volume of saline was intratracheally injected as a control. LiCl (F1409041, Aladdin reagent Co., LTD. Shanghai, China) was dissolved in sterile water, which was daily administered through intraperitoneal injection at a dose of 200 mg/kg/bw/day for 7 days since the second day after PPE injection. All of the mice were anaesthetized by intraperitoneal injection of 10% chloral hydrate (0.1 mL/kg) at day 21 post PPE exposure, and then, the animals were killed. In separate experiments, C57BL/6J mice were injected daily for 3 days with CSE (20%, 100 μL) through intratracheal instillation, and we treated these mice with LiCl (200 mg/kg, ip), metformin (250 mg/kg, oral gavage) or their vehicle (sterile water) 2 hours before CSE administration. Mice will be killed at 24 hours post‐CSE administration. Animal study was approved by the Anhui Medical University Animal Care Committee, and performed in accordance with the guidelines from the Care and Use of Laboratory Animals published in the NIH.

### 2.2 Histopathology and immunohistochemistry (IHC)

Mouse left lungs were inflated with 1% low melt agarose (SLBH4354V; sigma, USA) at a pressure of 25 cm H_2_O and then fixed with 4% neutral paraformaldehyde for 24 hours. Tissues were embedded in paraffin, sectioned (5 μm) and stained with haematoxylin and eosin (H&E). Mean linear intercept (Lm) was measured to show this verity of alveolar destruction as we previously described.^14^ Briefly, Lm was calculated in each sample based on 10 random fields and observed at a magnification of ×200 using a cross‐line. The sample slides were incubated with primary antibody against Mac‐3 (ab22506; Abcam, USA,1:1000) and Ly6G (sc‐168490; Santa Cruz, 1:250) for detecting of lung macrophages and neutrophils by IHC, respectively. After staining, the slides were examined under a light microscope by a photograph documentation facility (Olympus, Tokyo, Japan). Integrated optical density (IOD) of positive cells in each sample (at least 3 random microscopic fields per lung section, ×400) was analysed by Image pro‐plus 6.0 software.^15^

### 2.3 Cell culture and treatments

Human normal bronchial epithelial (NHBE) cells were obtained from the American Type Culture Collection (Cat#: CRL‐2078; Manassas, USA), and cultured in RPMI‐1640 media (Gibco, USA) supplemented with 15% foetal bovine serum (FBS; Gibco, USA), 100 U/mL penicillin and 100 U/mL streptomycin at 37^°^C in a humidified atmosphere containing 5% CO2. The cells were pretreated metformin (0.8 mmol/L, O0615AS; Meilun Biotech Co., Ltd, Dalian, China), human recombinant Wnt3a (rhWnt3a, 25 ng/mL, 5036‐WN‐010/CF; R&D, USA), or LiCl (10 mmol/L) for 24 hours before treatment with CSE.

### 2.4 Preparation of CSE

Research grade cigarette (3R4F, University of Kentucky, Lexington, KY) contains 17.1 mg of total particulate matter (TPM), 15 mg of tar and 1.16 mg of nicotine. CSE was extracted by bubbling smoke from one cigarette into 10 mL serum‐free media with a vacuum pump. CSE preparation was standardized by measuring the absorbance and adjusting to a pH of 7.45, which showed little variation between different batches of CSE. In the end, CSE was sterile filtered by 0.45 μm filter. We defined 100% CSE as one cigarette smoking in 10 ml medium with OD value 1.00 ± 0.05 at 320 nm wavelength.^16^, ^17^ CSE was freshly prepared, and diluted with culture media supplemented with 15% FBS immediately before use for experiments. Control medium was prepared at the same procedure.

### 2.5 Immunofluorescence

The cells were fixed with 4% paraformaldehyde for 20 minutes and then washed with PBS and permeabilization with 0.1% Triton ×100. After blocking with 5% BSA for 30 minutes at room temperature, the cells were incubated with the anti‐Nrf2 antibody (1:200) at 4°C overnight. Cells were rinsed with PBS for 3 times and added with FITC‐conjugated goat anti‐rabbit IgG (ZF‐0311; Beijing, China, 1:200) for 1 hour. 6‐diamidino‐2‐phenylindole (DAPI) (C1005; Beyotime Institute of Biotechnology, Shanghai, China) was employed to identify the nucleus. The images were captured by an Olympus UTBI90 Fluorescence microscope with the appropriate filters and identical acquisition parameters at ×200 magnification.

### 2.6 Statistical analysis

All the relevant data are expressed as mean ± SD. One‐way ANOVA following LSD test or Dunnett's T3 test were performed for multiple comparisons. The statistical analyses were conducted using the SPSS 13.0 software, and the values of *P* < .05 were considered to be statistically significant.

## 3 RESULTS

### 3.1 Wnt activation protected against PPE‐induced airspace enlargement and inflammatory cells influx in WT but not in Nrf2^−/−^ mice

**Figure 1:**
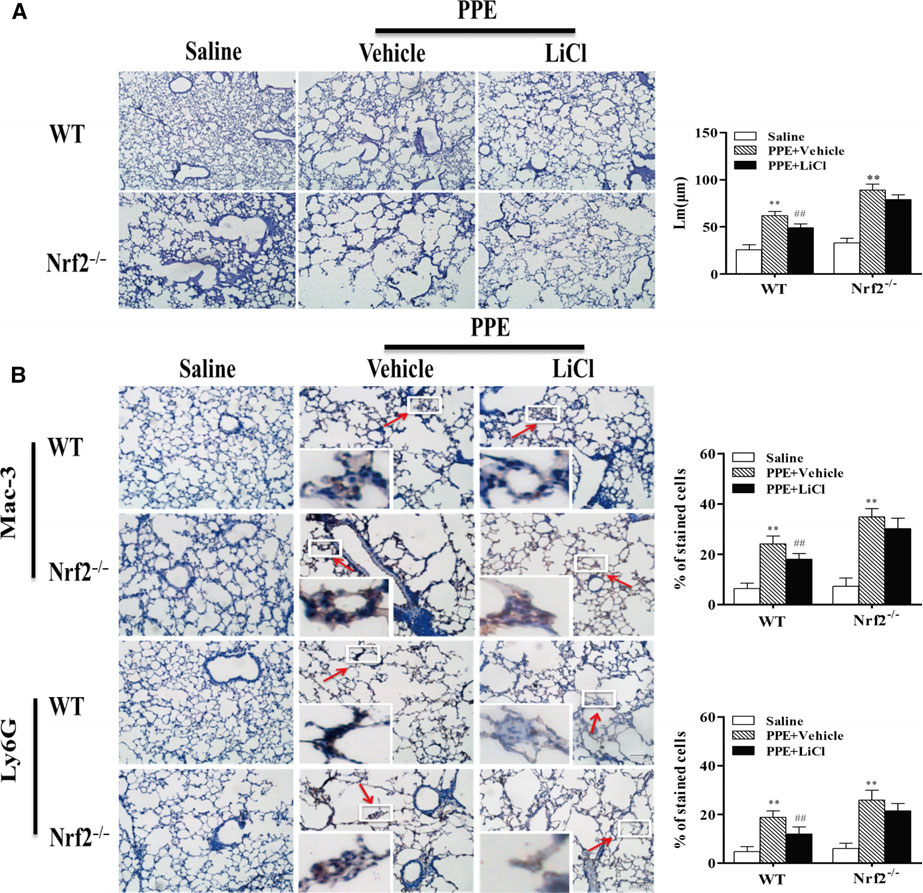
Wnt activation protected against PPE‐induced airspace enlargement and inflammatory cells influx in WT but not in Nrf2^−/−^mice. WT and Nrf2^−/−^ mice were subjected to PPE (80 U/kg, endotracheal injection) followed by LiCl treatment (ip, 200 mg/kg/bw/day). A, Lung sections from each mouse were stained with H&E, and the visualization of alveolar structure was examined with a light microscope ×200. B, Macrophages and neutrophils influx into mouse lungs were determined by IHC using Mac‐3 and Ly6G antibodies, respectively. The visualization was represented with a light microscope ×400. Histogram bars represent means ± S.D. (n = 3‐4 per group). **P < .01, vs the corresponding saline groups; ^##^ *P* < .01, vs the corresponding PPE‐exposed groups

To determine the role of Nrf2 in Wnt/β‐catenin‐mediated protection against emphysema, we treated WT and Nrf2^−/−^ mice with a Wnt signal activator (LiCl). Intratracheal injection of PPE caused the alveolar destruction (increased Lm), leading to emphysema in WT mice, which was further augmented in Nrf2^−/−^ mice (Figure 1A). Interestingly, LiCl treatment significantly decreased the damage of alveolar structure in WT but not Nrf2^−/−^ mice exposed to PPE. Next, we determined the infiltration of macrophages and neutrophils into lung interstitium of both WT and Nrf2^−/−^ mice through IHC staining. We found that the abundance of Mac‐3 and Ly6G was significant increased in lung tissues of both WT and Nrf2^−/−^ emphysematous mice (Figure 1B). These inflammatory cells infiltration were significant reduced by LiCl treatment in WT mice but not in Nrf2^−/−^ mice exposed to PPE (Figure 1B). These results demonstrate that LiCl treatment attenuates inflammatory responses and airspace enlargement in WT mice but not Nrf2^−/−^ mice.

### 3.2 Wnt activation increased Nrf2 levels and decreased IL‐6 protein in PPE‐induced WT but not Nrf2^−/−^ mice

**Figure 2:**
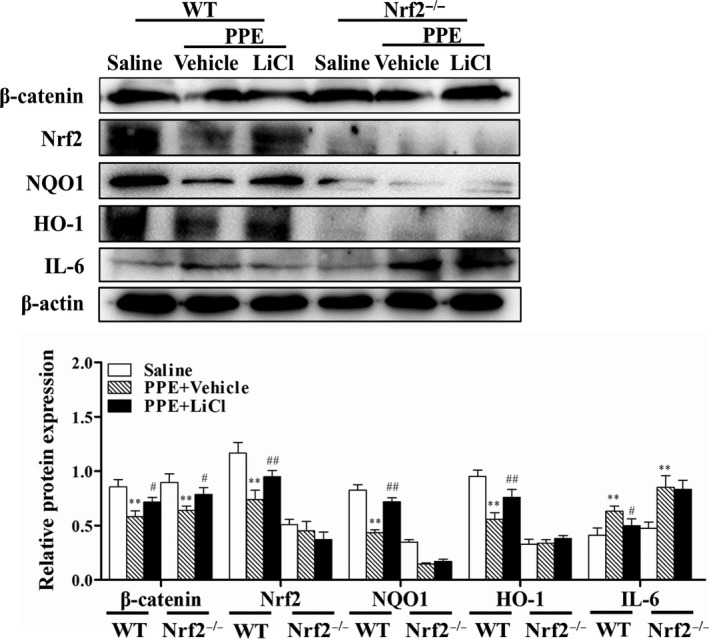
Wnt activation increased Nrf2 levels and decreased IL‐6 protein in PPE‐induced WT but not Nrf2^−/−^mice. Lung tissues from mice were subjected to Western blot for determining β‐catenin, Nrf2, HO‐1, NQO1 and IL‐6 levels. There presentative bands were obtained from different gels for repeated experiments. After densitometry, the values of proteins were normalized against β‐actin. Data were shown as mean ± SD (n = 3‐4 per group). **P < .01, vs the corresponding saline groups; ^#^ *P* < .05, ^##^ *P* < .01, vs the corresponding PPE‐exposed groups

The effect of LiCl on Nrf2 signal during PPE‐induced pulmonary emphysema was determined by western blot analysis. It revealed that PPE injection reduced β‐catenin and Nrf2‐dependent proteins HO‐1 and NQO1 (Figure 2). LiCl treatment increased levels of Nrf2, HO‐1 and NQO1 in WT, but not in Nrf2^−/−^ mice exposed to PPE. Similarly, LiCl reduced lung IL‐6 levels in WT but not in Nrf2^−/−^ mice exposed to PPE (Figure 2). However, the level of β‐catenin was not altered by Nrf2 deletion. These results suggest that Nrf2 mediates the protection of Wnt3a activation against PPE‐induced lung inflammatory responses.

### 3.3 Wnt activation decreased IL‐6 and KC levels in lung BAL fluid and homogenates in mice intratracheally injected with CSE

**Figure 3:**
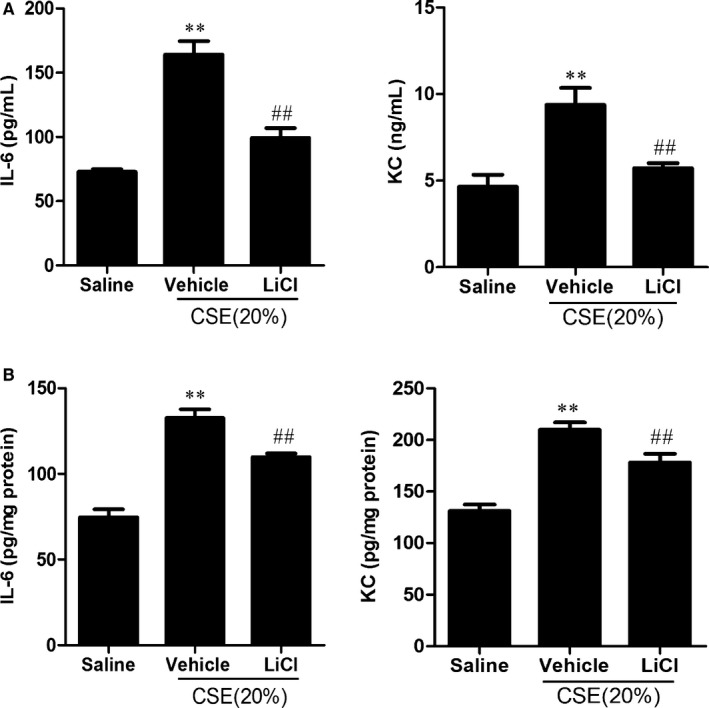
Wnt activation decreased IL‐6 and KC levels in lung BAL fluid and homogenates in mice intratracheally injected with CSE. Lung BAL fluid and homogenates were used for IL‐6 and KC detection by ELISA. Mice were intratracheally injected with CSE (20%, 100 μL) followed by LiCl treatment (ip, 200 mg/kg/bw/day). Lung BAL fluid (A) and homogenate (B) were used to detect IL‐6 and KC by ELISA. Histogram bars represent means± SD (n = 3‐4 per group). **P < .01, vs the corresponding saline groups; ^##^ *P* < .01, vs the corresponding CSE‐exposed groups

CS is a key factor for the development of lung inflammatory and injurious responses in COPD/emphysema. To determine whether Wnt signal modulates inflammatory responses to CS exposure, we administered CSE (20%) daily for 3 days through intratracheal injection into C57BL/6J mouse lung. CSE injection caused increase in IL‐6 and KC in lung BAL fluid and homogenates (Figure 3). Treatment with LiCl attenuated the levels of IL‐6 and KC in BAL fluid and lung homogenates in mice injected with CSE (Figure 3). These data suggest that LiCl reduces CS‐induced lung inflammation.

### 3.4 Wnt3a increased the Nrf2 activation and decreased IL‐6 levels in NHBE cells exposed to CSE

**Figure 4:**
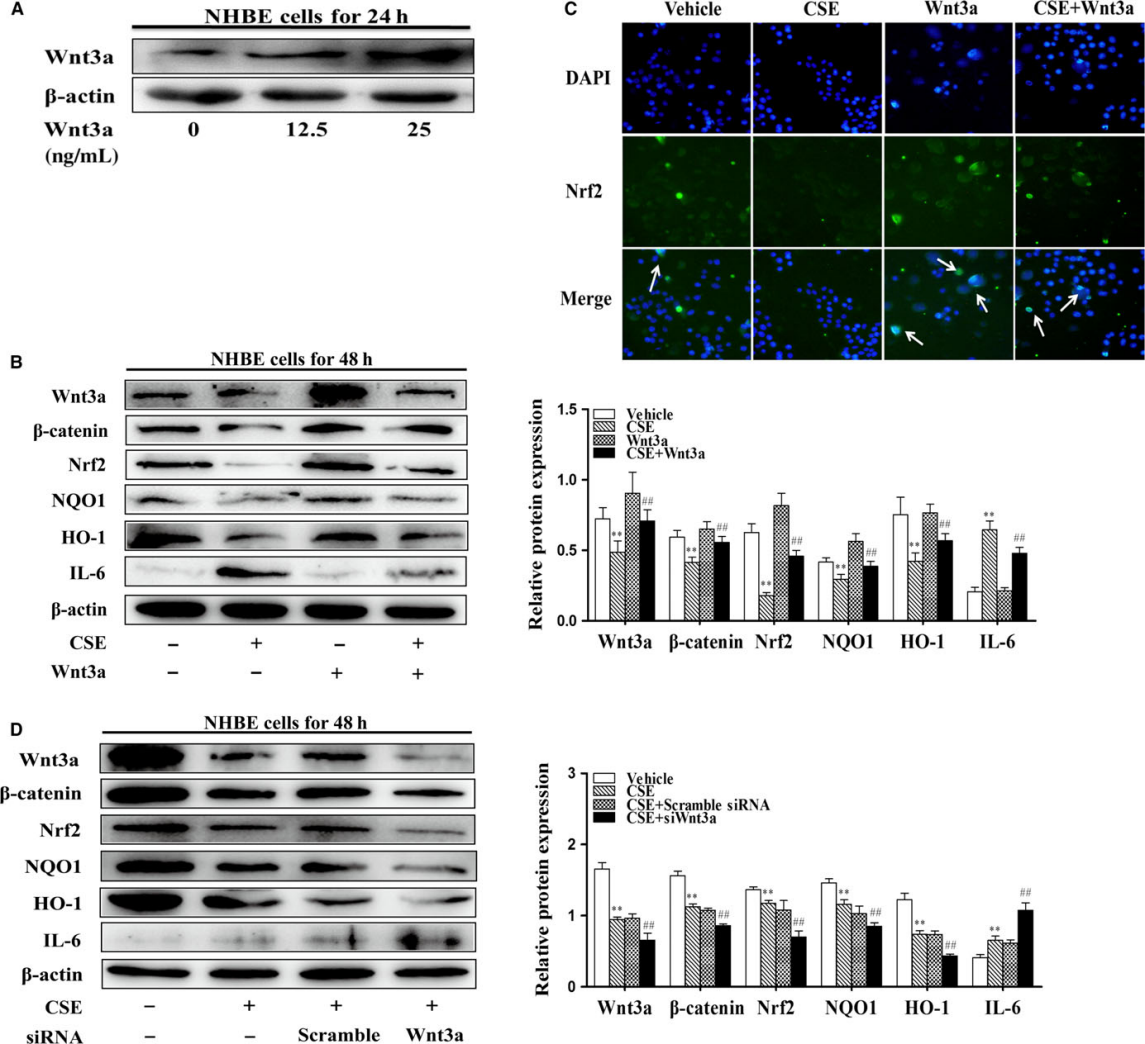
Wnt3a increased the Nrf2 activation and decreased IL‐6 levels in NHBE cells exposed to CSE. A, Different concentrations of rhWnt3a (0, 12.5, 25 ng/mL) were added to NHBE cells for 24 h. B, NHBE cells were treated with rhWnt3a (25 ng/mL) for 24 h before treated with CSE (1%) for 48 h. Related proteins of Wnt/β‐catenin and Nrf2 pathways: Wnt3a, β‐catenin, Nrf2, HO‐1, NQO1 and IL‐6 were detected by Western blot. C, Dual immunofluorescence in NHBE cells for Nrf2 (green) and nuclei (blue) was visualized by DAPI staining. White arrows indicated the nuclear translocation of Nrf2. Scale bars represent 100 μm. D, Wnt3a siRNA was transfected in NHBE cells before treatment with CSE (1%) for 48 h. Related proteins of Wnt/β‐catenin and Nrf2 pathways: Wnt3a, β‐catenin, Nrf2, HO‐1, NQO1 and IL‐6 were detected by Western blot. Representative bands were obtained from different gels for repeated experiments. After densitometry analysis, the values of proteins were normalized against β‐actin. Data were shown as mean ± SD (n = 3‐4 per group). **P < .01, vs the corresponding control groups; ^##^ *P* < .01, vs the corresponding CSE‐exposed groups

First, we assessed cell viability by MTT assay in response to various concentrations of CSE (0%‐4%) for 24 and 48 hours. Treatment with CSE higher than 1% significantly decreased cell viability in NHBE cells (Figure S1A). Hence, we investigated the effects of CSE (0, 0.25%, 0.5%, 1%) on the Wnt/β‐catenin and Nrf2 pathway proteins at 24 and 48 hours by Western blot. As shown in Figure S1B and C, the levels of Wnt3a, β‐catenin, Nrf2, HO‐1 and NQO1 were increased after CSE treatment for 24 hours, but reduced at 48 hours. Due to the highest changes in these proteins in NHBE cells treated with CSE (1%) for 48 hours, we utilized this CSE concentration and time in following experiments. Lung epithelium is one of the first targeting cells to CS. Therefore, we determined the regulation of Nrf2 by Wnt3a in NHBE cells exposed to CSE. Next, we determined the effects of Wnt3a on Nrf2 signals and IL‐6 levels using Wnt3a recombinant protein and knockdown approaches. Treatment with rhWnt3a (up to 25 ng/mL) for 24 hours increased their levels in NHBE cells (Figure 4A). As shown in Figure 4B, treatment with rhWnt3a (25 ng/mL) for 24 hours increased not only Wnt3a/β‐catenin but also Nrf2, HO‐1 and NQO1 levels by CSE treatment (1%) in NHBE cells. CSE‐induced increase in IL‐6 was significantly reduced by rhWnt3a (25 ng/mL). Immunofluorescence also showed the increasing nuclear translocation of Nrf2 after rhWnt3a treatment (Figure 4C). Furthermore, the responses observed in Figure 4B were reversed by Wnt3a siRNA transfection (Figure 4D). Nrf2 siRNA further increased IL‐6 levels by CSE. However, Nrf2 siRNA transfection had no effects on Wnt3a or β‐catenin protein in NHBE cells regardless of CSE exposure (Figure S2). Altogether, these findings further support the notion that Wnt3a activates Nrf2‐dependent signals but reduces IL‐6 levels in NHBE cells exposed to CSE, and Nrf2 protects against CS‐induced lung inflammatory responses but does not affect Wnt3a.

### 3.5 Wnt3a activation decreased IL‐6 level via Nrf2 in NHBE cells exposed to CSE

**Figure 5:**
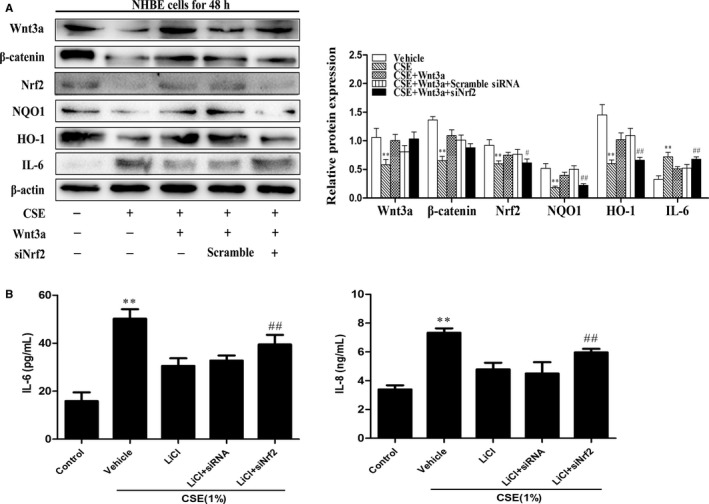
Wnt3a activation decreased IL‐6 level via Nrf2 in NHBE cells exposed to CSE. A, Nrf2 siRNA were transfected into NHBE cells for 6 h; then, the rhWnt3a (25 ng/mL) was added to cells for 24 h before treated with CSE (1%) for 48 h. Related proteins of Wnt/β‐catenin and Nrf2 pathways: Wnt3a, β‐catenin, Nrf2, HO‐1, NQO1 and IL‐6 were detected by Western blot. There presentative bands were from different gels for repeated experiments. After densitometry analysis, the values of proteins were normalized against β‐actin. B, Nrf2 siRNA were transfected into NHBE cells for 6 h; then, the LiCl (10 mmol/L, 24 h) was added to cells for 24 h before treated with CSE (1%) for 48 h. Level of pro‐inflammatory cytokines including IL‐6 and IL‐8 in cell supernatants was measured by ELISA. Data were shown as mean ± SD (n = 3‐4 per group). **P < .01, vs the corresponding control groups; ^#^ *P* < .05, ^##^ *P* < .01, vs the corresponding CSE+Wnt3a or CSE+LiCl group

To further determine whether Wnt3a reduces IL‐6 through Nrf2 signal, we treated with Wnt3a (25 ng/mL) for 24 hours in NHBE cells transfected with Nrf2 siRNA. As expected, rhWnt3a treatment increased the levels of Nrf2, HO‐1 and NQO1 but decreased IL‐6 levels (Figure 5A). In contrast, the effect of rhWnt3a on IL‐6, HO‐1 and NQO1 was attenuated in NHBE cells transfected with Nrf2 siRNA in response to CSE treatment (Figure 5A). Similarly, Nrf2 knockdown by siRNA transfection abolished the protection of LiCl (10 mmol/L, 24 hours) against CSE (1%)‐induced increase in IL‐6 and IL‐8 release from NHBE cells (Figure 5B). These results suggest that Wnt3a reduces IL‐6 by activating Nrf2 in NHBE cells.

### 3.6 AMPK reduction in lungs of PPE‐induced emphysematous mice, and metformin decreased IL‐6 and IL‐8 levels via Nrf2 in NHBE cells exposed to CSE

**Figure 6:**
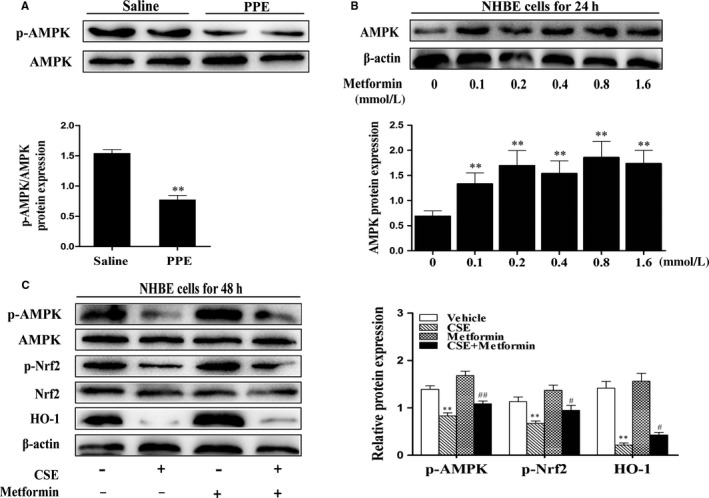
AMPK reduction in lungs of PPE‐induced emphysematous mice, and AMPK increased p‐Nrf2 level in NHBE cells exposed to CSE. A, Mice were subjected to PPE (80 U/kg, endotracheal injection). Lung tissues from mouse were subjected to Western blot analysis for AMPK, p‐AMPK. B, NHBE cells were treated with different concentrations of metformin (0, 0.1, 0.2, 0.4, 0.8, 1.6 mmol/L) for 24 h. C, Metformin (0.8 mmol/L) was given to NHBE cells for 24 h before treated with CSE (1%) for 48 h. Related proteins: p‐AMPK, p‐Nrf2, HO‐1 and β‐catenin were detected by Western blot. Presentative bands were obtained from different gels for repeated experiments. Data were shown as mean ±SD (n = 3‐4 per group). **P < .01, vs the corresponding control groups; ^#^ *P* < .05, ^##^ *P* < .01, vs the corresponding CSE‐exposed groups

AMPK facilitates Nrf2 phosphorylation and nuclear translocation for antioxidant response element (ARE)‐driven gene transactivation.^11^, ^18^ Hence, we determined phosphorylation of AMPK (p‐AMPK) in emphysematous lungs induced by PPE by Western blot. As shown in Figure 6A, the level of p‐AMPK was significantly reduced in lung of emphysematous mice. To further determine whether AMPK plays an important role in Nrf2‐dependent signals, NHBE cells were pretreated with a pharmacological activator of AMPK metformin prior to CSE treatment (1%) for 48 hours. As shown in Figure 6B, treatment with metformin (0‐1.6 mmol/L) for 24 hours significantly increased the level of AMPK in NHBE cells. CSE‐induced reduction in p‐AMPK, p‐Nrf2, β‐catenin and Nrf2 target gene product HO‐1 was significantly increased after metformin (0.8 mmol/L) treatment for 24 hours in NHBE cells (Figure 6C).

**Figure 7:**
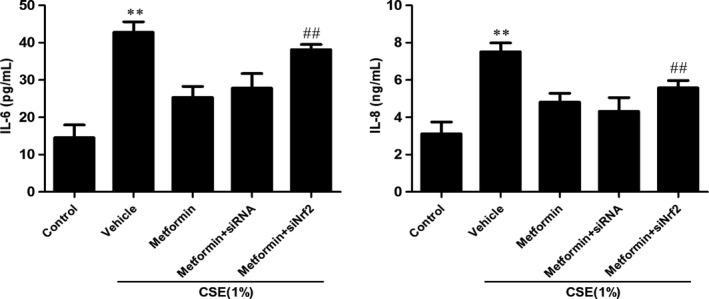
Metformin decreased IL‐6 and IL‐8 levels via Nrf2 in NHBE cells exposed to CSE. Nrf2 siRNA were transfected into NHBE cells for 6 h, and then, metformin (0.8 mmol/L) was added to cells for 24 h before treated with CSE (1%*)* for 48 h. Level of pro‐inflammatory cytokines IL‐6 and IL‐8 in cell supernatants were measured by ELISA. Data were shown as mean±SD (n = 3‐4 per group). **P < .01, vs the corresponding control groups; ^##^ *P* < .01, vs the corresponding CSE+Metformin group

Next, we determined whether activated AMPK attenuates inflammatory responses via Nrf2. We treated metformin (0.8 mmol/L) for 24 hours in NHBE cells transfected with Nrf2 siRNA in response to CSE (1%) incubation. Metformin significantly decreased IL‐6 and IL‐8 levels determined by ELISA in scramble siRNA transfected NHBE cells exposed to CSE (Figure 7). However, these protective effects of metformin were abolished in NHBE cells transfected with Nrf2 siRNA (Figure 7). These results suggest that Nrf2 partially mediates the protective role of AMPK against CSE‐induced inflammatory responses in human lung epithelial cells.

### 3.7 Metformin decreased IL‐6 and KC levels in lung BAL fluid and homogenates in mice intratracheally injected with CSE

**Figure 8:**
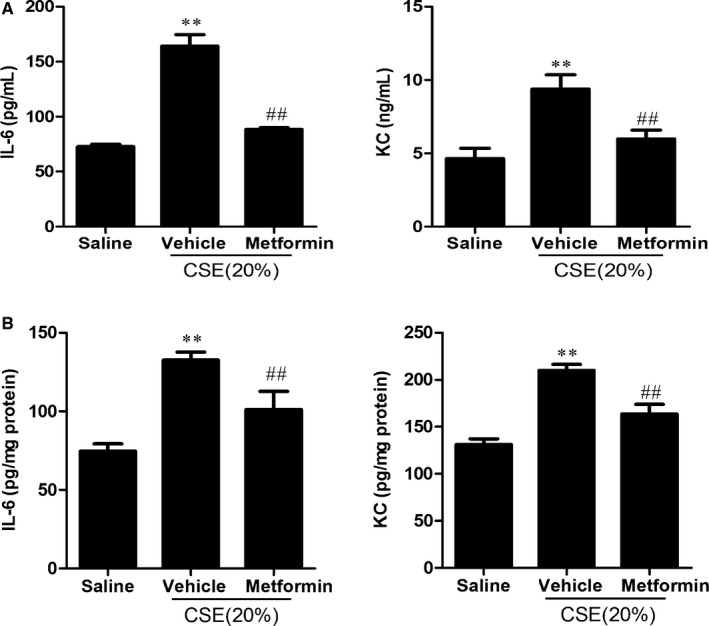
Metformin decreased IL‐6 and KC levels in lung BAL fluid and homogenates in mice intratracheally injected with CSE. Lung BAL fluid (A) and homogenates (B) were used for detecting IL‐6 and KC by ELISA. Mice were intratracheally injected with CSE (20%, 100 μL) followed by metformin treatment (250 mg/kg, oral gavage). Histogram bars represent means±SD (n = 3‐4per group). **P < .01, vs the corresponding saline groups; ^##^ *P* < .01, vs the corresponding CSE‐exposed groups

Although we have shown that AMPK attenuates PPE‐induced emphysema,^19^ it remains elusive whether metformin decreases CS‐induced inflammatory response in mouse lungs. We administered metformin (250 mg/kg, oral gavage) to C57BL/6J mice 2 hours before intratracheal injection of CSE (20%, 100 μL) daily for 3 days. We found that metformin significantly reduced CSE‐induced increase in IL‐6 and KC levels in BAL fluid and homogenates in mice (Figure 8). These results suggest that AMPK activation reduces lung inflammatory responses to CS.

### 3.8 Metformin increased the Nrf2 activation via Wnt3a/β‐catenin pathway in NHBE cells exposed to CSE

**Figure 9:**
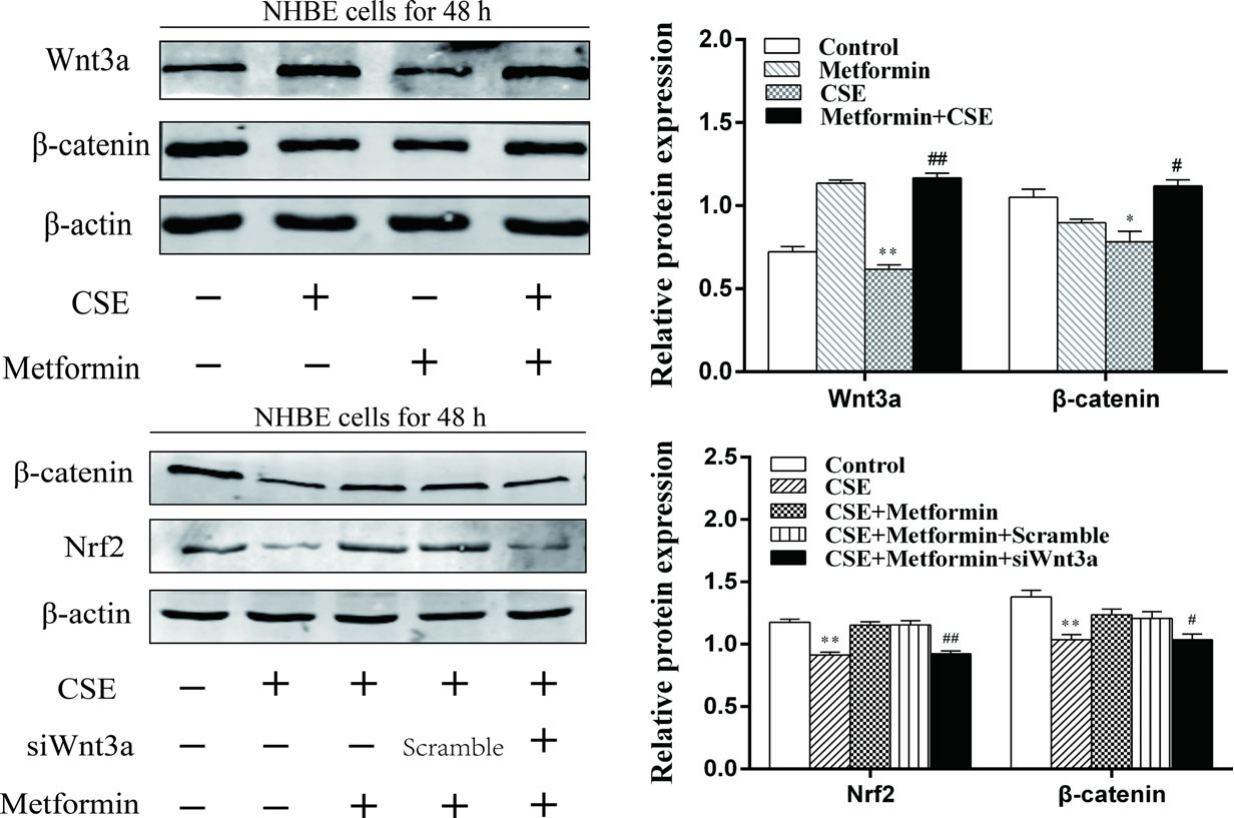
Metformin increased the Nrf2 activation via Wnt3a/β‐catenin pathway in NHBE cells exposed to CSE. A, Metformin (0.8 mmol/L) was given to NHBE cells for 24 h before treated with CSE (1%) for 48 h. Related proteins: Wnt3a and β‐catenin were detected by Western blot. B, Wnt3a siRNA were transfected into NHBE cells for 6 h; then, the metformin (0.8 mmol/L) was added to cells for 24 h before treated with CSE (1%) for 48 h. Proteins of Wnt3a, β‐catenin and Nrf2 were detected by Western blot. Representative bands were obtained from different gels for repeated experiments. Data were shown as mean ± SD (n = 3‐4 per group). *P < .05, **P < .01, vs the corresponding control groups; ^*#*^ *P* < .05, ^*##*^ *P* < .01, vs the corresponding CSE‐exposed groups

To further determine the relationship between AMPK and Wnt3a/β‐catenin pathways to the effect of Nrf2 activation, we treated metformin (0.8 mmol/L) for 24 hours in CSE‐exposed NHBE cells. These results showed that metformin significantly increased Wnt3a and β‐catenin levels (Figure 9A). However, the effects of metformin on Nrf2 and β‐catenin were attenuated in NHBE cells transfected with Wnt3a siRNA (Figure 9B). Altogether, these date suggest that AMPK partially effects the activation of Nrf2 and CSE‐induced lung inflammation, at least in part, mediated by increasing Wnt3a/β‐catenin pathway activity.

## 4 DISCUSSION

The pathogenesis of COPD comprises several major cellular events, including inflammatory responses, oxidative stress, protease/antiprotease imbalance and cellular senescence.^2^, ^20^ COPD is a complex disease with progressive destruction of functional lung tissue with few effective therapies.^1^, ^21^ These highlights the need for searching new molecular targets and active pathways involved in the progression of this disease. Here, we reported that activation of Wnt pathway by LiCl and AMPK by metformin protected against lung inflammatory responses and airspace enlargement, which is mediated by Nrf2 signal.

In addition to Keap1, Nrf2 can be subjected to SCF/β‐TrCP‐dependent ubiquitination and proteasomal degradation in a GSK3β‐dependent but β‐catenin‐independent manner.^10^, ^22^ GSK‐3β is one of the β‐catenin destruction complexes in Wnt pathway as the previously described.^5^, ^23^ Although Wnt/β‐catenin pathway has been shown to protect against the development of COPD/emphysema,^5^, ^24^ it remains unknown whether these protective effects are mediated by Nrf2. Our findings have shown that Wnt activation by LiCl attenuated elastase‐induced emphysema and CS‐induced inflammatory responses, which was not observed in Nrf2^−/−^ mice. This suggests that Nrf2, at least partially, mediates the protective role of Wnt activation against the development of emphysema. Although elastase‐induced lung injury is a reasonable model of emphysema,^25^, ^26^ future study using CS exposure in mice would further confirm the role of Wnt/Nrf2 axis in development of emphysema. In the present study, we only measured the levels of IL‐6 and KC cytokines in mouse lungs, the determination of other cytokines, such as CXCL‐1, TNF‐α, MMP‐9, using the Luminex Multiplex assays in future studies would unravel the changes in pro‐inflammatory mediator profile. It is also unknown whether LiCl‐dependent GSK3β inhibition causes the interaction between β‐catenin and Nrf2 in elastase instillation and CS exposure, which needs further investigation.

Wnt signalling is complex including several components. The intracellular Wnt signalling has at least 2 kinds: the canonical Wnt pathway (β‐catenin‐dependent) and the non‐canonical Wnt signalling including Wnt/Ca^2+^ pathway. Non‐canonical Wnt signalling is independent of β‐catenin transcriptional function.Wnt3a major locates in the bronchial and alveolar epithelial cells, which mediates the canonical Wnt pathway^27^ and has been reported to potently stimulate β‐catenin‐dependent Wnt signalling in vitro. Wnt5a is expressed in both epithelium and mesenchyme in the lung and is involved in non‐canonical Wnt signalling.^28^, ^29^ Wnt3a and Wnt5a have shown to drive either pro‐ or anti‐inflammatory responses in different diseases. For example, reduced Wn3a but increased Wnt5a were observed in rat lungs under endotoxin insult.^30^ Further study to determine the expression of Wnt5a during the development of emphysema would differentiate roles of Wnt3a and Wnt5a. It is also interesting to note that Wnt3a/β‐catenin proteins were increased in NHBE cells treated with CSE for 24 hours but was reduced by CSE treatment for 48 hours. This may be due to the posttranslational modification leading to their degradation by longer treatment of CSE, which needs further investigation.

AMPK is a cellular metabolic sensor which is activated by an increase in the intracellular AMP/ATP ratio and other cellular stresses.^31^, ^32^ AMPK acts on a wide variety of substrates and coordinate multiple pathways involved with metabolism and inflammatory responses.^33^, ^34^ It has been shown that AMPK can activate Nrf2 by promoting Nrf2 phosphorylation and nuclear translocation.^11^ We found that AMPK was de‐activated in mouse lungs with emphysema and CSE‐treated NHBE cells. Although we and others have shown that AMPK protects against lung inflammatory responses and airspace enlargement using pharmacological and genetic approaches,^19^, ^35^ it is not known whether this protection is mediated by Nrf2. Using Nrf2 siRNA in NHBE cells, we found that Nrf2 deficiency diminished the protection of metformin against CSE‐induced pro‐inflammatory mediator release. This suggests that AMPK protects against CS‐induced inflammatory responses by activating Nrf2 pathway. Nevertheless, the treatment of metformin in Nrf2^−/−^ mice is required to determine whether Nrf2 is needed for the protection of AMPK against CS‐induced emphysema.

Upon translocated into nucleus, Nrf2 binds to the ARE in the upstream promoter region of many anti‐oxidative genes (eg, NQO1, HO‐1, glutathione S‐transferase) and initiates their transcription. This explains that major function of Nrf2 is to protect against oxidative stress by inducing the production of anti‐oxidants. We found increased inflammatory responses in Nrf2 KO mice exposed to elastase or CSE‐treated cells transfected with Nrf2 siRNA. This may be due to increased oxidative stress in Nrf2 deficient cells which leads to increased inflammatory responses to CS.

AMPK crosstalks with Wnt signal in regulating cell functions.^36^, ^37^, ^38^, ^39^ For example, AMPK can phosphorylate β‐catenin at Ser 552, which stabilizes β‐catenin and enhances β‐catenin/TCF mediated transcription.^40^ GSK3, an important functional protein of Wnt pathway, is also linked to AMPK. A recent study revealed that GSK3 inhibits AMPK function though forming a stable complex with AMPK regulatory subunit AMPK β and catalytic subunit AMPK α.^38^ In our study, we found that AMPK activation by metformin significantly increased the level of WNT pathway ligand protein Wnt3a. Nevertheless, it is unknown whether AMPK competes with Wnt ligands or GSK3β in phosphorylating β‐catenin thereby modulating Wnt signal during the development of emphysema.

CS exposure only reproduces the mild aspects of emphysema but requires 4‐6 months of duration.^41^ Therefore, we utilized the elastase‐induced emphysema model, where lung injury develops rapidly and severely. This is relatively easy (one time injection) and cheap (do not need smoke machine/facility) to assess the effects of LiCl on the development of emphysema.^26^ Nevertheless, future studies using CS exposure model would provide more convincing data regarding the role of Wnt3a and AMPK on airspace enlargement and lung function decline in COPD/emphysema.

**Figure 10:**
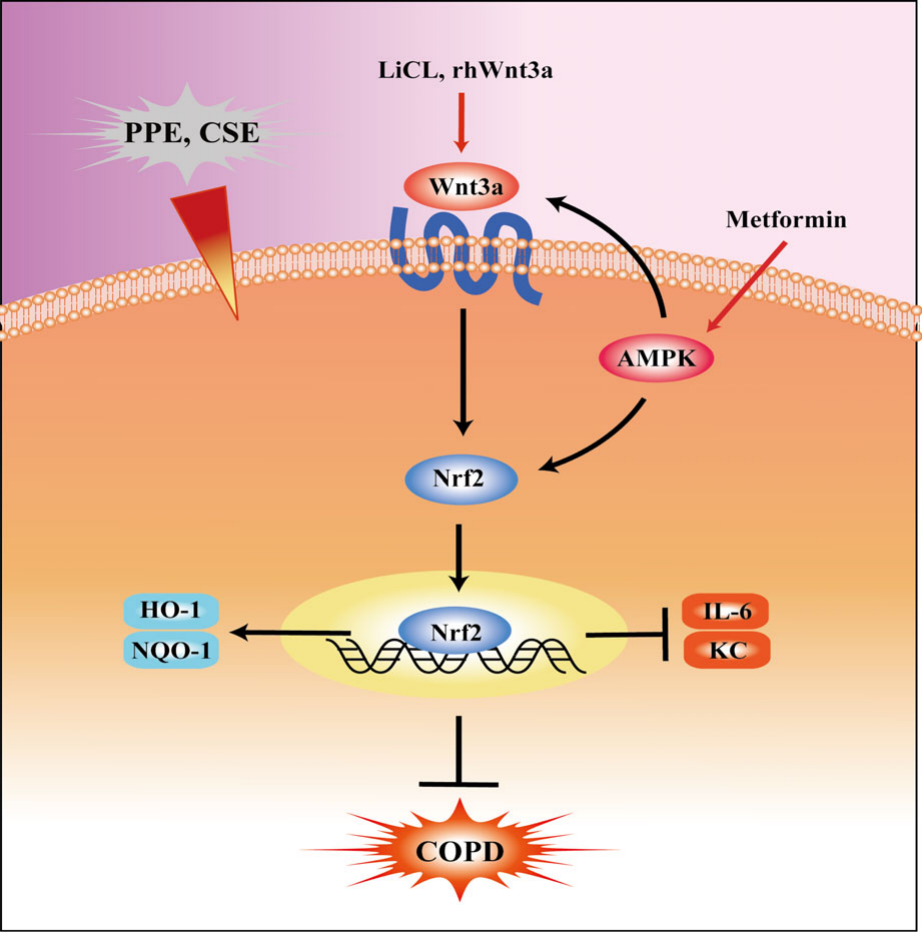
Schematic overview of Nrf2 signalling crosstalk to both Wnt3a/β‐catenin and AMPK pathway in COPD/emphysema

In conclusion, we demonstrated that Wnt3a/β‐catenin signalling selectively suppressed the expression of the pro‐inflammatory responses and airspace enlargement by regulating Nrf2 pathway. AMPK could promote the activation of Wnt3a/β‐catenin and Nrf2 pathways but reduced inflammatory responses to CS exposure. Altogether, Nrf2 as a downstream signal of both AMPK and Wnt pathways attenuated lung inflammatory responses and emphysema (Figure 10). These findings provide new therapeutic possibilities by targeting Wnt3a/β‐catenin and AMPK pathways, as well as activating Nrf2 in treatment of COPD/emphysema.

## CONFLICT OF INTEREST

The authors confirm that there are no conflicts of interest.
