# Supplementary figures and images for: Nrf2 attenuates inflammatory response in COPD/emphysema: Crosstalk with Wnt3a/β‐catenin and AMPK pathways
Source: J Cell Mol Med. 2018 Apr 16;22(7):3514–25. doi: 10.1111/jcmm.13628 (PMC6010849; doi:10.1111/jcmm.13628)

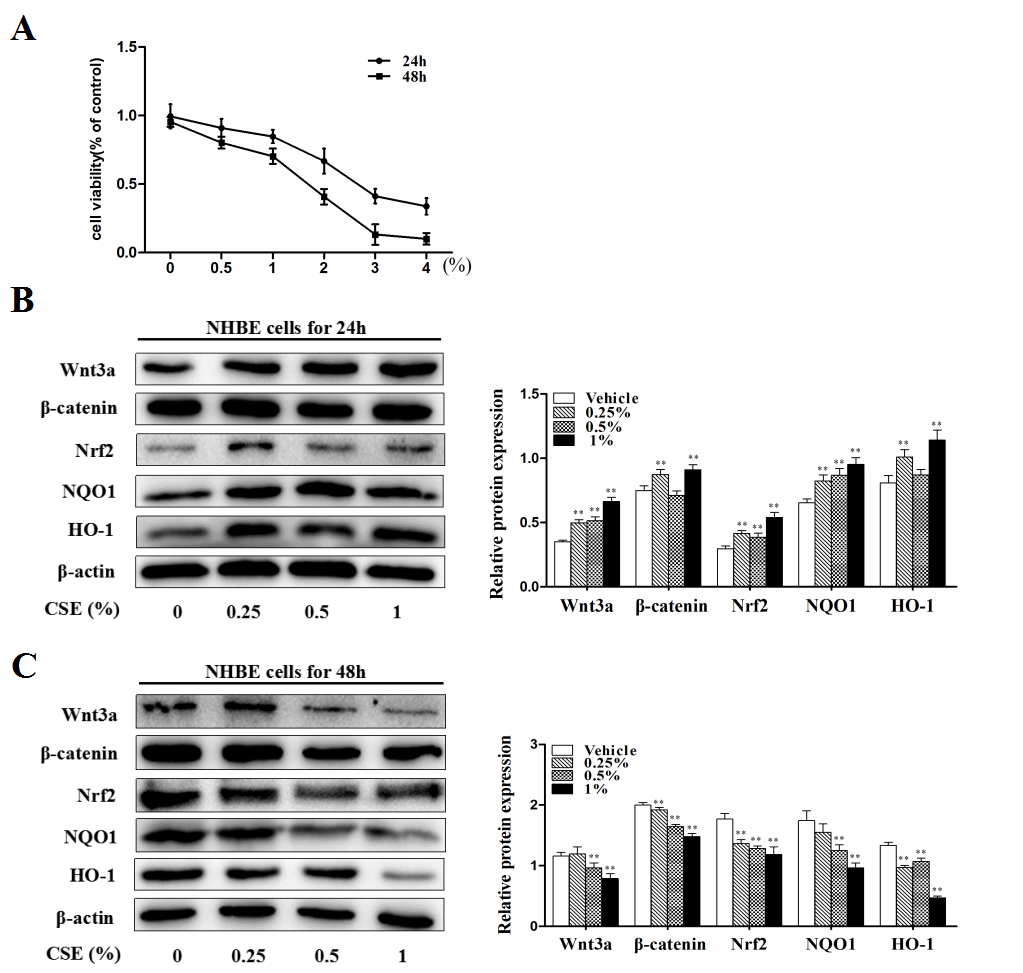

Supplement: Supplementary file 1 [file JCMM-22-3514-s001.tif]

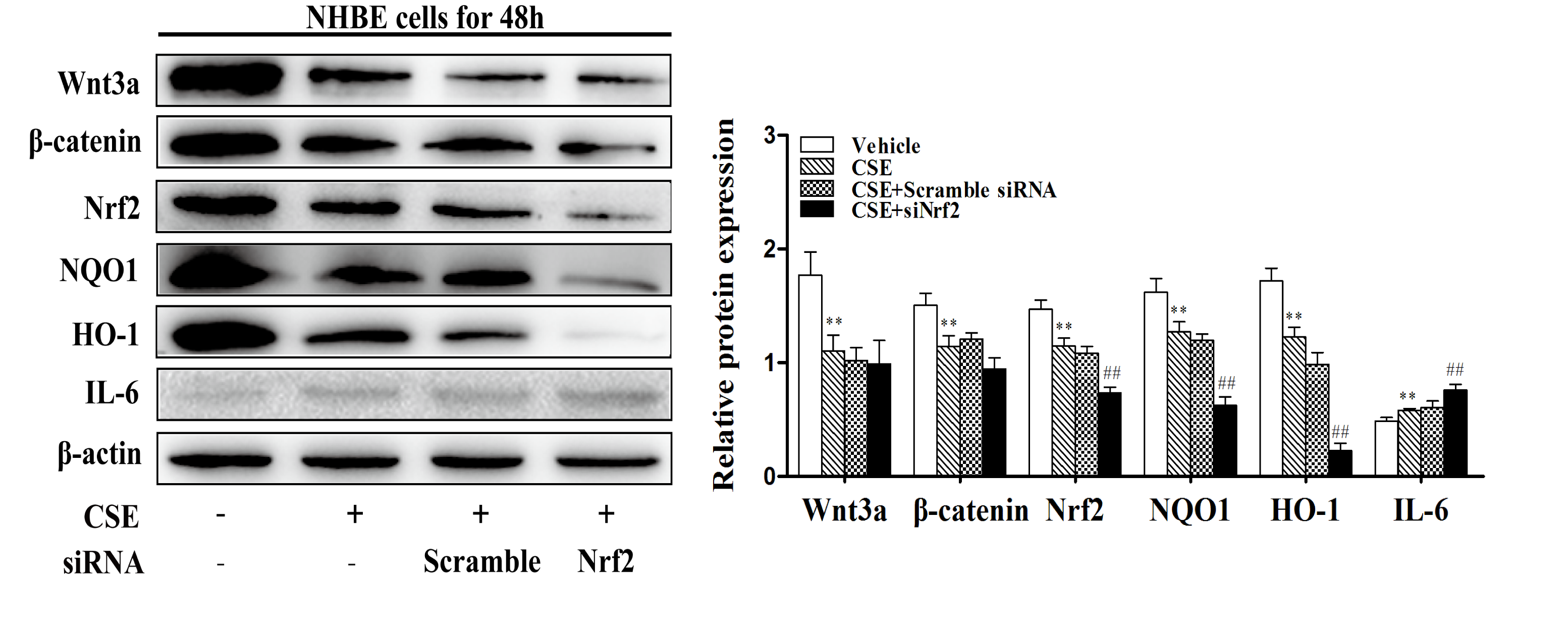

Supplement: Supplementary file 2 [file JCMM-22-3514-s002.tif]
