# Supplementary material for: Nrf2 attenuates inflammatory response in COPD/emphysema: Crosstalk with Wnt3a/β‐catenin and AMPK pathways
Source: J Cell Mol Med. 2018 Apr 16;22(7):3514–25. doi: 10.1111/jcmm.13628 (PMC6010849; doi:10.1111/jcmm.13628)
Supplement: Supplementary file 3 [file JCMM-22-3514-s003.docx]

**SUPPORTING INFORMATION**

**Manuscript #: JCMM-04-2017-029 Revision #3**

**TITLE: Nrf2 attenuates inflammatory response in COPD/emphysema: crosstalk with Wnt3a/β-catenin and AMPK pathways**

Wenhui Cui^1, 2, 3, #^, Zhihui Zhang^1, 2, #^, Panpan Zhang^4, #^, Jiao Qu^4^, Cheng Zheng^1^, Xiaoting Mo^1^, Wencheng Zhou^1^, Liang Xu^1^, Hongwei Yao^5, *^, Jian Gao^1, 2, *^

^1^ First Affiliated Hospital of Anhui Medical University, Hefei, Anhui, China;

^2^ Second Affiliated Hospital of Dalian Medical University, Dalian, Liaoning, China;

^3^ General Hospital of Datong Coal Mining Group, Datong, Shanxi, China;

^4^ School of Pharmacy, Dalian Medical University, Dalian, Liaoning, China;

^5^ Department of Critical Care and Pulmonary Medicine, Shanxi Medical University Second Hospital, Taiyuan, Shanxi 030001, P.R. China.

**Materials and Methods**

**Bronchoalveolar lavage (BAL) fluid and lung homogenates**

Right lungs were lavaged with 0.6 ml PBS each time four times. BAL fluid was collected and centrifuged at 3,000 rpm for 10 min at 4°C, the supernatant of the lavage fluid was stored at -80°C for cytokine analysis. Right lung lobes (60–100 mg) were homogenized with 1:10 (wt/vol) of cool saline, and the homogenate was centrifuged at 3000 rpm for 10 min at 4°C, the supernatant of lung homogenate was stored at -80°C for further analysis.

**Western blot**

Lung tissues and the cells were collected and homogenized by a lysis buffer (P0013C; Beyotime Institute of Biotechnology, Shanghai, China) containing the proteinase inhibitor PMSF (Amresco 0754; Biosharp, USA). The mixture was centrifuged at 12,000 rpm for 10 min at 4^0^C, and the supernatant was collected and stored in -80^0^C. Protein concentrations in the supernatant of the lysed sample were determined by the BCA method. The equivalent amount of protein samples was separated using 12% SDS-PAGE gel, which was transferred to PVDF membranes (20130107054; Millipore, USA). After blocking with 5% nonfat milk (Guangming, China) in TBST for 2 h, the membranes were incubated with primary antibodies including anti-Wnt3a (ab28472; Abcam, USA, 1:1000), anti-β-catenin (ab32572; Abcam, USA, 1:500), anti-AMPK (#2532; Cell Signaling, USA, 1:1000), anti-p-AMPK (ab195946; Abcam, USA, 1:1000), anti-p-Nrf2 (ab76026; Abcam, USA, 1:1000), anti-Nrf2 (ab31163; Abcam, USA, 1:800), anti-HO-1 (ab13243; Abcam, USA, 1:2000), anti-NQO1 (bs-2184R; Bioss, Beijing, China, 1:200), anti-IL-6 (BS6419; Bioworld, Shanghai, China; 1:1000), or anti-β-actin (ab52614; Abcam, USA, 1:5000) antibodies at 4^0^C overnight. Peroxidase conjugated secondary antibodies as goat anti-rabbit lgG (ZB-2301; ZSGB-BIO, Beijing, China, 1:5000) in 5% nonfat milk were incubated for 1 h. Finally, the signals were imaged by enhanced chemiluminescence reagent (ECL; Thermo Scientific, Rockford, USA). The densitometry of bands was analyzed by the Image J software.

**Cytokine measurement**

The concentrations of IL-6 and KC in the supernatants of lavage fluid, lung homogenate and cell culture were quantified by an enzyme-linked immunosorbant assay (ELISA) according to the manufacturer’s instructions. The kits of human IL-6 (CK-E10140) and IL-8 (CK-E95353), mouse IL-6 (CK-E20012) and KC (CK-E94862) were obtained from the Nanjing Jiancheng Bioengineering Institute, Nanjing, China. The OD values were read at 450 nm wavelength, and the real values of IL-6, KC and IL-8 were plotted based on their standard curves.

**MTT assay**

Cell viability was examined by MTT assay. The NHBE cells were seeded on 96-well plates at a density of 1.5×104 cells/well, and were maintained in normal culture until 70% confluence. Then the cells were stimulated by different concentrations of CSE (0, 0.5%, 1%, 2%, 3%, 4%) for 24 h or 48 h. Cells were then incubated with 20 µl MTT solution (5 mg/ml phosphate buffer; Sigma, USA) at 370C for 4h, then solubilized with 150 µl DMSO (Sigma, USA) at room temperature for 10 min. The absorbance at 490 nm was detected using a microplate reader (Multiskan MK3; Thermo Scientific, USA), and cell viability was expressed as a percentage of the control culture value.

**Transfections**

Wnt3a small interfering RNA (siWnt3a), siNrf2, and negative control (scramble) siRNA were synthesized according to human-specific sequences (GenePharma, Shanghai, China). The sequences of these siRNAs were as follows: Wnt3a, the forward primer was 5’-CCAUGAACCGCCACAACAATT-3’ and the reverse was 5’-UUGUUGUGGCGGUUCAUGGTT-3’. Nrf2, the forward primer was 5’-GCACCUUAUAUCUCGAAGUTT-3’, and the reverse was 5’-ACUUCGAGAUAUAAGGUGCTT-3’. These siRNAs were transfected into NHBE cells with Lipofectamine 2000 (Invitrogen, USA) in Opti-MEM serum free medium (Gibco, USA) in 6-well plates according to the manufacturer’s instructions. Briefly, we mixed 200 μl of FBS-free medium and 8 μl of Lipofectamine 2000 as solution A, and 200 μl FBS-free medium and 8 μl targeted or scramble siRNA as solution B. After 5 minutes, we mixed solution A and solution B, and allow them to form complex for 20 min. Finally, the mixture was added into the 6-well plate containing 1.6 ml FBS-free medium each well. After 6-hour culture, we replaced FBS-free medium by new medium containing 15% FBS.

**FIGURE LEGENDS**

**Supplemental Figure 1. Doses of CSE treatment to detect Wnt/β-catenin and Nrf2 pathways for 24 h and 48 h in NHBE cells.** (A) NHBE cells were exposed to various concentrations of CSE (0, 0.5%, 1%, 2%, 3%, 4%) for 24 h or 48 h. Viable cells were detected by MTT assay. (B-C) Effects of CSE (0, 0.25%, 0.5%,1%) on the Wnt/β-catenin and Nrf2 pathway proteins: Wnt3a, β-catenin, Nrf2, HO-1 and NQO1 at 24 h or 48 h, which were detected by Western blot. These presentative bands were obtained from different gels for repeated experiments. After densitometric analysis, the values of proteins were normalized against β-actin. Data were shown as mean±S.D. (n=3-4 per group). **P<0.05, **P<0.01* vs the corresponding Control groups.

**Supplemental Figure 2. Nrf2 knockdown increased IL-6 levels but had no effects on Wnt signals in NHBE cells exposed to CSE**

Nrf2 siRNA was transfected in NHBE cells before treated with CSE (1%) for 48h. Related proteins of Wnt/β-catenin and Nrf2 pathways: Wnt3a, β-catenin, Nrf2, HO-1, NQO1 and IL-6 were detected by Western blot. These presentative bands were obtained from different gels for repeated experiments. After densitometry analysis, the values of proteins were normalized against β-actin. Data were shown as mean±S.D. (n=3-4 per group). **P<0.05, **P<0.01*, vs the corresponding Control groups;*^#^P<0.05, ^##^P<0.01,* vs the corresponding CSE-exposed groups.
